# Fecal microbiota transplantation and its repercussions in patients with melanoma refractory to anti-PD-1 therapy: scope review

**DOI:** 10.1590/0100-6991e-20233490-en

**Published:** 2023-05-04

**Authors:** EDUARDO CERCHI BARBOSA, EDUARDA EMÍLIA CRUZ BUCAR, GABRIEL RODRIGUES JUBÉ, LETÍCIA BONFIM SILVEIRA, NATÁLIA CÂNDIDO DUAILIBE SILVA, PEDRO CARVALHO CAMPOS FARIA, PEDRO LUCAS CARNEIRO RAMOS, VITOR RYUITI YAMAMOTO MORAES, JOÃO ORMINDO BELTRÃO BARROS

**Affiliations:** 1 - Universidade Evangélica de Goiás, Medicina - Anápolis - GO - Brasil; 2 - Hospital Santa Casa de Anápolis, Cancerologia Cirúrgica - Anápolis - GO - Brasil

**Keywords:** Antibodies, Monoclonal, Immunotherapy, Melanoma, Microbiota, Fecal Microbiota Transplantation, Anticorpos Monoclonais, Imunoterapia, Melanoma, Microbiota, Transplante de Microbiota Fecal

## Abstract

**Introduction:**

despite being extremely effective in some cases, up to 70% of patients with melanoma do not respond to anti-PD-1/PD-L1 (primary resistance) and many of the responders eventually progress (secondary resistance). Extensive efforts are being made to overcome this resistance through new strategies, especially aimed at modulating the intestinal microbiota*.*

**Objective:**

to assess whether fecal microbiota transplantation (FMT), associated with immunotherapy, is beneficial in the clinical course of patients with refractory melanoma.

**Methods:**

this is a scope review, based on studies collected on the MEDLINE, ScienceDirect, The Cochrane Library, Embase and BMJ Journals; using the terms: “Antibodies, Monoclonal”; “Drug Resistance, Neoplasm”; “Fecal Microbiota Transplantation”; “Host Microbial Interactions”; “Immunotherapy”; “Melanoma”; and “Microbiota”. Clinical trials, in English, with relevant data on the subject and fully available were included. A cut-off period was not determined, due to the limited amount of evidence on the topic.

**Results:**

crossing the descriptors allowed the identification of 342 publications and, after applying the eligibility criteria, allowed the selection of 4 studies. From the analyses, it was observed that a considerable part of those studied overcame resistance to immune checkpoint inhibitors after FMT, with better response to treatment, less tumor growth and increased beneficial immune response.

**Conclusion:**

it is noted that FMT favors the response of melanoma to immunotherapy, translated into significant clinical benefit. However, further studies are necessary for the complete elucidation of the bacteria and the mechanisms involved, as well as for the translation of new evidence to oncological care practice.

## RESUMO

**Introdução:**

apesar de extremamente eficaz em alguns casos, até 70% dos pacientes com melanoma não respondem aos anti-PD-1/PD-L1 (resistência primária) e muitos dos respondedores, eventualmente, acabam progredindo (resistência secundária). Extensos esforços estão sendo realizados para superar esta resistência através de novas estratégias, sobretudo, visando a modulação da microbiota intestinal.

**Objetivo:**

avaliar se o transplante de microbiota fecal (TMF), associado à imunoterapia, é benéfico no curso clínico do paciente com melanoma refratário.

**Métodos:**

trata-se de uma revisão de escopo, baseada em estudos coletados nas plataformas MEDLINE, ScienceDirect, The Cochrane Library, Embase e BMJ Journals; utilizando os descritores: “Antibodies, Monoclonal”; “Drug Resistance, Neoplasm”; “Fecal Microbiota Transplantation”; “Host Microbial Interactions”; “Immunotherapy”; “Melanoma”; e “Microbiota”. Foram incluídos ensaios clínicos, na língua inglesa, com dados relevantes sobre a temática e disponíveis integralmente. Não foi determinado um período de corte temporal, devido à quantidade limitada de evidências sobre o tema.

**Resultados:**

o cruzamento dos descritores permitiu a identificação de 342 publicações e, após a aplicação dos critérios de elegibilidade, permitiu a seleção de 4 estudos. A partir das análises, observou-se que grande parte dos estudados superaram a resistência aos inibidores do checkpoint imunológico pós-TMF, com melhor resposta ao tratamento, menor crescimento tumoral e aumento da resposta imunológica benéfica.

**Conclusão:**

nota-se que o TMF favorece a resposta do melanoma à imunoterapia, traduzido por benefício clínico significativo. Entretanto, novos estudos são necessários para a completa elucidação das bactérias e mecanismos envolvidos, bem como para que haja a translação das novas evidências para a prática assistencial oncológica.

## INTRODUCTION

Cancer is a state that involves both genetic and epigenetic interactions, culminating in a disorderly, autonomous, and uncontrollable proliferation of cells, which may eventually migrate to adjacent structures. This migratory growth characteristic belongs to invasive neoplasms, being called “malignant tumors”. There are more than 200 types of cancer, each with specific clinical and morphological characteristics, with melanoma being the third most common and the most lethal among skin cancers, with an estimated increase of 50% in new cases per year by 2040, according to data from the International Agency for Research on Cancer (IARC)^1^ ^,^ ^2^.

Melanoma is a neoplasm originating from the skin formed from the transformation of melanin-producing cells (melanocytes) and may appear in skin without previous lesions (60-80%) or with pigmented lesions (20-40%), such as melanocytic nevi^3^. Although it has a lower incidence when compared with non-melanomas, melanoma is the most important form of skin tumor, as it grows and spreads more quickly, has a higher risk of recurrence, and a worse prognosis, with a worldwide survival estimate in five years of 69%, 73% in developed countries and 56% in developing ones^4^ ^,^ ^5^.

Among the risk factors for the development of this tumor, the inheritance of sun-sensitive skin stands out (Fitzpatrick skin types I and II - light skin, hair, and eyes, and susceptibility to sunburn). Other factors include the presence of pigmented lesions, such as freckles, atypical nevi, or large numbers of common nevi (more than 50), intermittent sun exposure, sunburn (especially during childhood), artificial ultraviolet radiation (eg, use of tanning beds), proximity to the equator, and previous melanoma^6^ ^,^ ^7^. Positive family history for both melanoma and multiple atypical nevi is also considered a relevant factor. Mutations in the CDKN2A and CDK4 genes have been detected in some families with hereditary melanoma, conferring an approximately 60-90% increased risk for this neoplasm^8^ ^,^ ^4^. In addition, the immune status also seems to influence the development of melanoma, as this neoplasm is frequent in immunocompromised patients due to organ transplantation, human immunodeficiency virus (HIV) infection, hematologic malignancy, or use of immunosuppressants^9^.

The diagnostic suspicion, in general, is based on the identification of a new pigmented lesion (ab initio) or phenotypic alterations of a pre-existing melanocytic nevus. During the assessment, the mnemonic “ABCDE” has been shown to be useful: A - asymmetry; B - irregular edges; C - non-uniform coloring; D - diameter > 6mm; E - evolution, including rapid growth, pruritus, ulceration, and bleeding. The greater the positivity of these signs, the greater the probability of the lesion being a malignant melanoma. However, the definitive diagnosis is made through histopathological examination, which can identify the superficial disseminated, nodular, acral lentiginous, or lentigo maligna patterns^10^ ^,^ ^11^.

In terms of prognosis and treatment, staging the disease is essential, in accordance with the American Joint Commission on Cancer (AJCC) protocol, which divides patients into four distinct groups. The group is defined according to the phase the patient is in, in which stages I and II are considered clinically localized disease, stage III, locoregional disease, including individuals with metastases to regional or in-transit lymph nodes, and stage IV, comprising individuals with distant metastasis^12^.

After histopathological confirmation and staging, the treatment to be adopted is established^13^. The therapeutic management of melanoma includes different approaches, such as surgical intervention (excision with widened margins, sentinel lymph node investigation, lymphatic drainage, and resection of distant metastases), adjuvant treatment, systemic therapy, and/or radiotherapy^14^.

In cases of unresectable, metastatic cutaneous melanoma or with high rates of recurrence (stages III and IV), specific therapies should be considered, such as immunotherapy with immune checkpoint inhibitors (ICIs). Currently, the drugs most used in this treatment are the humanized monoclonal antibodies (mAbs) anti-PD-1 (pembrolizumab and nivolumab) and anti-PD-L1 (atezolizumab, avelumab, and durvalumab), which act by inhibiting the activity of the programmed cell death protein-1 (PD-1)^15^ ^,^ ^16^. These drugs bind to the PD-1 receptor, an immunological receptor present on the surface of tumor cells, preventing the receptor from interacting with specific ligands (PD-L1 and PD-L2). Blocking the PD-1 pathway signaling cascade inhibits the negative regulation of the immune system, reversing the suppression of T cells and culminating in the anti-tumor response^17^.

Anti-PD-1/PD-L1 demonstrated superior efficacy and a more favorable toxicity profile than other mAbs, such as ipilimumab, an inhibitor of cytotoxic T lymphocyte-associated antigen 4 (CTLA-4), also widely used in the therapeutic approach of melanoma^18^ ^,^ ^19^. However, although these blockers show extreme effectiveness in some cases, up to 70% of patients do not respond to PD-1 blockade (primary resistance) and many who do respond eventually progress (secondary resistance). Therefore, efforts are currently being made to understand in detail the mechanisms underlying resistance, aiming to elucidate biomarkers that can potentially help in identifying responders, as well as finding new strategies that can decrease anti-PD-1/PD-L1 resistance^20^ ^-^ ^24^.

In search of advances in medicine, researchers returned to a Chinese practice dating back to the fourth century, during the Dong Jin dynasty, known as fecal microbiota transplantation (FMT). This practice consists of transferring fecal material from a healthy donor to a sick recipient, to repopulate the latter’s intestine. Despite being very old, FMT was scientifically reported for the first time in 1958, when it was successfully used in the treatment of four patients with pseudomembranous colitis^25^ ^-^ ^27^. Even in the face of apparent efficacy, only in the last ten years has FMT begun to be widely studied, having proved to be beneficial, mainly when associated with other therapies, in various pathologies, including cancers such as gastric, colorectal, hepatocellular, pancreatic, breast, lung, and melanoma^28^ ^-^ ^33^.

Therefore, due to the favorable pre-existing history, the present study aims to evaluate whether the ICI-FMT combined therapy positively impacts the clinical course of patients with melanoma refractory to isolated immunotherapy, representing a potential step during the therapeutic process of this neoplasm.

## METHODS

This is a descriptive study of exploratory nature in literature in the scope review modality, which is a research method that makes it possible to identify and analyze a set of scientific evidence to obtain a reliable understanding about a particular theme of study. This method makes it possible to summarize the state of knowledge in a given area, as well as pointing out gaps that need to be filled with new research. Therefore, it entails support for decision-making and improvement of clinical practice^34^.

We used the Preferred Reporting Items for Systematic Reviews and Meta-Analyses Extension for Scoping Reviews (PRISMA-ScR)^34^ strategy, through the following steps: (i) theme identification; (ii) formulation of the research question; (iii) collection of articles through searches on electronic platforms; (iv) eligibility of studies with application of inclusion and exclusion criteria; (v) assessment of the quality of evidence; (vi) elaboration of the collection instrument with the information to be extracted; (vii) data analysis, interpretation, synthesis, and discussion; and (viii) presentation of results. The final protocol was prospectively registered in the Open Science Framework platform.

The research question was formulated based on the PICO strategy (Population, Intervention, Control, Outcome)^35^, where: Population - patients with melanoma refractory to immunotherapy with ICIs; Intervention - FMT + ICI; Control - ICIs monotherapy; Outcome - clinical benefit. Thus, we formulated the following guiding question: “Does the combined ICI-FMT therapy have a positive impact on the clinical course of patients with refractory melanoma?”. To answer this question, we carried out a broad search, aiming to minimize publication biases, in the following databases, in order of consultation: Medical Literature Analysis and Retrieval System Online (MEDLINE); ScienceDirect; The Cochrane Library; Excerpta Medica Database (EMBASE); and BMJ Journals. During the search, we used the following Health Sciences Descriptors: “Antibodies, Monoclonal”; “Drug Resistance, Neoplasm”; “Fecal Microbiota Transplantation”; “Host Microbial Interactions”; “Immunotherapy”; “Melanoma”; and “Microbiota”.

The inclusion criteria of the studies were clinical trials with relevant data on the application of ICI-FMT therapy in patients with melanoma, research written only in English, and articles with the text available in full. We excluded articles outside thematic relevance and non-clinical studies, including research in animal and in vitro models, as well as those published in the form of letters to the editor, guidelines, books, literature reviews, dissertations, and case reports. A time cut-off period was not determined during the selection of studies, due to the limited amount of evidence on the subject.

## RESULTS

The combination of search terms resulted in the identification of 342 publications. After removal of duplicates (n=27), 315 references were submitted to screening, with analysis of titles and abstracts, of which 302 were excluded by reviewers. The 13 remaining studies were read in full by two independent examiners for the application of the eligibility criteria, culminating in the final inclusion of four trials that met these criteria. The selected studies were published between 2019 and 2021.

After conducting the steps of identification, screening, and eligibility of the articles, it was possible to stratify and gather the main evidence related to the application of the ICI-FMT combined therapy in patients with melanoma. Studies extracted from each of the databases were recorded in a single Microsoft Excel 16.0 spreadsheet, with the aim of detecting repeated citations between platforms, forming the list of studies and assessing eligibility.

The results of clinical trials that investigated the impact of FMT on the effectiveness of the immune checkpoint inhibitor are summarized in Table 1.

**Table 1:** Selected clinical trials on the application of FMT associated with ICI in patients with refractory melanoma.

Type of FMT-associated immunotherapy	Type of cancer	Number of patients studied	Number of patients with greater efficacy (%)	Response to FMT in patients with greater efficacy	Reference
Anti-PD-1	Metastatic melanoma	5	3 (60%)	Increased infiltration of functional CD8+ T cells; increase in APCs (CD68+) in intestine and tumor; resistance to immunotherapy overcome	Youngster et al. (2019)^36^
Anti-PD-1	Metastatic melanoma	2	2 (100%)	Increase in functional CD39+ and CD8+ T cells; decrease in dysfunctional PD1+, CD38+ and CD8+ T cells; resistance to immunotherapy overcome	Maleki et al. (2020)^37^
Anti-PD-1	Metastatic melanoma	15	6 (40%)	Higher number of functional CD8+ T cells; decrease in IL-8 producing myeloid cells; resistance to immunotherapy overcome	Davar et al. (2021)^38^
Anti-PD-1	Metastatic melanoma	10	3 (30%)	Increased infiltration of functional CD8+ T cells; increase in APCs (CD68+) in intestine and tumor; resistance to immunotherapy overcome	Baruch et al. (2021)^39^

*APCs: antigen-presenting cells. Source: Authors (2023).*

## DISCUSSION

### PD-1/PD-L1 pathway as a therapeutic target

PD-1 is a protein of the CD28 superfamily, characterized by generating negative signals when binding to PD-L1 and PD-L2 proteins. Both PD-1 and its ligands are widely expressed in different cell types (B cells, CD4+ and T CD8+ cells, natural killer cells, and dendritic cells) that physiologically act by limiting the activation and proliferation of T cells, as well as promoting immunological tolerance to self-antigens, preventing auto-inflammatory reactions and autoimmunity in the healthy host^40^ ^,^ ^41^.

However, after continuous exposure and recognition of tumor antigens, tumor-specific effector T cells induce increased expression of PD-1 and secrete interferon-gamma (IFN-γ), a chemical signal that induces the expression of B7 H1 (PD-L1) in neoplastic cells. The PD-1/PD L1 interaction strongly suppresses T cell activation, disrupting its anti-tumor activity. This interruption of the T cell antineoplastic response, related to the anergy or “exhaustion” phenotype, represents a form of local immunocompromise that allows tumors to escape immunological surveillance^42^ ^,^ ^43^.

**Figure 1:**
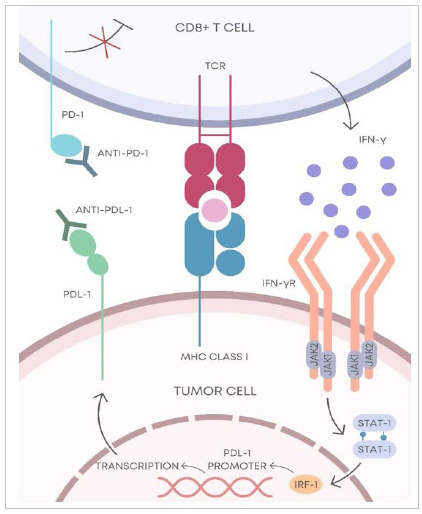
PD-1/PD-L1 pathway blocking mechanism. The CD8+ T cell is activated after recognizing the tumor antigen presented by the MHC class I. After its activation, the CD8+ T cell releases IFN-γ, which binds to its receptor (IFN γR). From this connection, the tumor cell is induced to express PD-L1, which binds to the PD-1 protein, triggering an inhibitory effect against CD8+ T cells. Anti-PD 1 or anti PD L1 prevents the interaction between PD 1 and PD L1, suppressing the inhibitory action against CD8+ T cells and thus increasing the anti-tumor activity. PD-1, programmed cell death 1; anti-PD-1, PD-1 antibody; PDL-1, programmed-death ligand 1; anti-PDL-1, PDL-1 antibody; TCR, T cell receptor; MHC, major histocompatibility complex; IFN-γ; interferon-γ; IFN-γR, IFN- γ receptor; JAK1, janus kinase 1; JAK2, janus kinase 2; STAT-1, signal transducer and activator of transcription 1; IRF-1, interferon regulatory factor 1. Source: Adapted from Lei et al. (2020) ^24^ .

Blocking the PD-1/PD-L1 pathway (Figure 1) is considered one of the main targets of immunotherapy against cancer, with drugs already approved by the Food and Drug Administration (FDA), such as pembrolizumab, nivolumab, atezolizumab, and cemiplimab^24^ ^,^ ^44^ ^-^ ^47^. Table 2 highlights each drug, with its respective indications and main adverse effects.

**Table 2:** Applications and adverse effects of the main anti-PD-1/PD-L1 immunotherapies.

References	Antibody / drug	Indications	ADEs
Garon et al. (2015)^43^ Martin-Liberal, et al. (2015)^48^ Ribas et al. (2016)^49^ Springman et al. (2020)^50^	Anti-PD-1 Pembrolizumab	Melanoma, NSCLC, TCC, gastric cancer, cHL, RCC, HNSCC, esophageal cancer, CRC, endometrial cancer, HCC	Fatigue, cough, nausea, pruritus, skin rash, decreased appetite, cold, arthralgia, diarrhea, infections
Robert et al. (2015)^45^ Guo, Zhang, Chen (2017)^51^ Elias et al. (2017)^52^ Springman et al. (2020)^50^	Anti-PD-1 Nivolumab	Melanoma, NSCLC, TCC, gastric cancer, cHL, RCC, HNSCC, esophageal cancer, HCC, MPM	Skin rash, fatigue, dyspnoea, myalgia, decreased appetite, cough, nausea, cold
Schmid et al. (2018)^46^ Elias et al. (2017)^52^ Tie et al. (2019)^53^	Anti-PD-L1 Atezolizumab	Breast cancer, NSCLC, TCC	Fatigue, decreased appetite, dyspnoea, cough, nausea, myalgia, cold, urinary infection, hypothyroidism, alopecia
Migden et al. (2018)47 Goodman (2022)54 Sezer et al. (2021)55	Anti-PD-1 Cemiplimab	BCC, SCC	Diarrhea, fatigue, nausea, cold, chills, cough, diarrhea, pyrexia, hoarseness, pruritus, skin rash, backache

*ADEs: adverse drug effects; NSCLC: non-small cell lung carcinoma; TCC: transitional cell carcinoma; cHL: classic Hodgkin’s lymphoma; RCC: renal cell carcinoma; HNSCC: head and neck squamous cell cancer; CRC: colorectal cancer; HCC: hepatocellular carcinoma; MPM: malignant pleural mesothelioma; BCC: basal cell carcinoma; SCC: squamous cell carcinoma. Source: Authors (2023).*

However, clinical data of anti-PD-1/PD L1 therapy show considerable rates of limited response, in which a large group of patients suffered primary resistance, not responding to therapy, and part of the responders developed acquired resistance over the course of treatment^43^. Extensive efforts are being made to overcome this resistance to therapy, especially in the approach to melanoma^20^ ^-2^ ^4^.

### Intestinal Microbiota

The intestinal microbiota, previously referred to as flora, is a dynamic mixture of microorganisms, whose composition varies along the gastrointestinal tract (GIT). The GIT hosts the largest number and diversity of bacterial collections that colonize the human body. It is estimated that its microbial population reaches 10^11^ to 10^12^ CFU/mL of intestinal contents, with about 700 different species of microorganisms, most of which are bacteria. However, although bacteria can be found throughout the GIT, the largest number resides in the colon^56^ ^,^ ^57^.

In the ileum, the bacterial population is represented by 10^7^ to 10^8^ CFU/mL, consisting of facultative anaerobes, Enterobacteria, and obligate anaerobes, such as *Veilonella, Enterococcus, Clostridium, Bacteroides*, and *Lactobacillus*. On the other hand, in the large intestine, the population reaches the 10^10^ and 10^11^ CFU/mL and the most frequently found genera are *Bacteroides, Bifidobacterium, Escherichia coli, Bacillus, Clostridium, Eubacterium, Fusobacterium, Peptostreptococcus,* and *Ruminococcus* ^56^ ^-^ ^58^.

As a rule, facultative anaerobic bacteria such as *E. coli, Enterococci faecalis,* and *E. faecium* are the first to colonize the newborn’s GIT, due to the high level of oxygen that initially exists. As these microorganisms consume the gas, the medium becomes more suitable for restricted anaerobic bacteria such as *Bacteriodes, Bifidobacterium,* and *Clostridium*. The intestinal microbiota acquired in the postnatal period is composed of a vast diversity of bacteria and performs numerous functions in the human host^56^.

Among its various functions, the immunological stands out. The human intestinal mucosa is the main interface between the immune system and the external environment. The intestine is considered the largest immunological organ in the human body, housing about 80% of the cells of this system, being mainly responsible for the production of immunoglobulins essential for innate and adaptive immunity. In addition, it stimulates the immune system by recruiting immune cells and activating the function of epithelial cells, thus being able to modulate the organism’s multiple response pathways and tumor progression itself^56^ ^,^ ^59^ ^-^ ^62^.

Moreover, the intestinal microbiota also influences the effectiveness of numerous drugs. With regard to antineoplastic agents, one of the first drugs to prove the influence of the intestinal microbiota on its response was cyclophosphamide. With the advancement of high-throughput sequencing technologies, the importance of the intestinal microbiota in the modulation of chemotherapy drugs was listed and the search for new influenceable drugs is now increasingly recognized^63^ ^,^ ^64^.

However, the microbiota is extremely dynamic and may be influenced by numerous factors. For example, most neoplasms reach extremes of age, when the structural ecology of the gut may be immature or disturbed by a lifetime’s exposure to environmental modifiers^65^. Furthermore, pathological states or specific therapies can also create an imbalance in the microbiota composition, increasing the influence of deleterious bacteria, as well as reducing the effectiveness of antineoplastic agents and exacerbating their toxicity. Therefore, the intestinal microbiota has been researched for the management of cancer, with the development of promising strategies, such as the modulation of the patients’ intestinal microorganisms’ content^28^ ^-^ ^33^.

### Fecal microbiota transplantation

FMT consists of transferring a solution of fecal material from a donor to a recipient, aiming to directly change the microbial composition of the patient and confer benefits to his health. The first known report of the use of feces as a therapy was described by Ge Hong, in China, in the fourth century, for the treatment of a variety of conditions, including diarrhea^27^ ^,^ ^66^ ^,^ ^67^. However, it was only in the 1950s that the practice was scientifically described by Eiseman et al. (1958)^25^, via fecal enema, for the treatment of pseudomembranous colitis, introducing FMT in conventional medicine.

In general, the process initially involves selecting a donor with no personal or family history of metabolic, autoimmune, or malignant diseases, as well as screening for possible pathogens. The stool is then prepared by being mixed with water or normal saline, followed by a filtration step to remove any particulate matter. The mixture can be administered by nasogastric tube, nasojejunal tube, esophagogastroduodenoscopy, colonoscopy, retention enema, or lyophilized tablets. The choice of route depends on the institution facility, the physician’s experience, and the safety offered to the patient^68^.

Most of the clinical experience with FMT has been derived from the treatment of recurrent or refractory *Clostridium difficile* infection^66^ ^,^ ^68^. However, recent studies have listed its benefits, especially when associated with other therapies, in several pathologies, including cancers, such as gastric, colorectal, hepatocellular, pancreatic, breast, lung, and melanoma^28^ ^-^ ^33^.

### Influence of FMT on the response to anti-PD-1/PD-L1 in mice

Given the evidence that the intestinal microbiota influenced the response to chemotherapy^63^ ^,^ ^64^, tests were carried out in mice with melanoma that would later be treated with FMT associated with immunotherapy. In the study by Sivan et al. (2015)^69^, two groups of genetically similar C57BL/6 mice from different locations were studied, the JAX group from the Jackson Laboratory and the TAC group from Taconic Farms, both of which presented differences in the intestinal microbial composition. Initially, melanoma cells were injected into both groups, and, before any other intervention, tumor growth was analyzed, which was more aggressive in TAC mice compared with JAX. Such responses were explained by divergences at the immunological level, the JAX group exhibiting a greater density of specific T cells against the tumor and a greater intratumoral accumulation of CD8+ T cells. After receiving treatment with PD-1 pathway inhibitors, significantly greater efficacy was observed in JAX mice.

Subsequently, to establish a cause-effect relationship, the fecal material of one of the JAX mice was transferred to TAC ones, generating a reduction in the tumor growth rate. In addition, some TAC mice were transplanted with JAX fecal microbiota associated with an anti-PD-L1, showing an even more effective response in tumor control. The analysis of the fecal material verified the predominance of bacteria of the genus *Bifidobacterium (B. longum, B. breve,* and *B. adolescentis)*, which were found to be four hundred times more abundant in JAX mice. The presence of Bifidobacterium was strictly related to specific immune cytotoxicity against the tumor, translated by an exacerbated regulation of type-I IFN genes in antigen presenting cells (APCs) present in peripheral lymphoid organs, as well as a higher rate of maturation of dendritic cells and increased activity of effector CD8+ T cells^69^.

Based on the study by Sivan et al. (2015)^69^, new research was carried out aiming to list the differences in the constitution of the intestinal microbiota between individuals responsive (R) and non-responsive (NR) to immunotherapy, such as the studies by Frankel et al. (2017)^70^ and McCulloch et al. (2022)^71^. The former used a technique for sequencing the intestinal components of 39 individuals with metastatic melanoma before starting therapy with ICIs (ipilimumab, nivolumab, and pembrolizumab, either in monotherapy or combined). In general, all the R presented a microbiota enriched in *Bacteroides caccae* and *Streptococcus parasanguinis*, compared with the NR. In the second study, the analysis of the composition of the microbiota of 94 patients allowed the identification of the predominance of the following bacteria in group R: *Ruminococcus (Mediterraneibacter) torques, Blautia (B. producta, B. wexlerae,* and *B. hansenii), Eubacterium rectale, Ruminococcus (Mediterraneibacter) gnavus,* and *Anaerostipes hadrus*. On the other hand, in patients not responsive to anti-PD 1, a predominance of *Prevotella* spp., *Oscillibacter* spp., *Alistipes* spp., and *Sutterellaceae* spp. was observed. Subsequently, the fecal samples from these patients were taken for transcriptomic analysis, identifying a considerable increase of superoxide dismutase 2 (SOD2), pro-inflammatory cytokines (IL-1β and CXCL8), and transcription factors (NFKBIZ, NFKBIA, TNFAIP3, and LITAF) in NR.

Aiming once again to determine the differences in intestinal microbial composition between immunotherapy responders and non-responders, Gopalakrishnan et al. (2018)^72^ showed that, in the R group (complete or partial response or stable disease for at least six months), there was a predominance of Faecalibacterium (belonging to the *Ruminococcaceae* family, order *Clostridiales*), while in the NR group (progressive or stable disease for less of six months), there was a predominance of *Bacteroides thetaiotaomicron*, *Escherichia coli,* and *Anaerotruncus colihominis*. In view of the results, the possible mechanisms by which the microbiota patterns could influence the response were described: in R patients, bacteria mainly exerted anabolic functions and biosynthesis of amino acids, in contrast to NR, in which predominantly catabolic functions were observed. Furthermore, in the R group, there was a greater infiltration of CD8+ T cells in the tumor, as well as a greater diversity of immune cells. In order to confirm this relationship, fecal material from group R was transferred to germ-free mice, followed by inoculation of neoplastic melanoma cells and administration of PD 1 inhibitor. After two weeks, the transplanted mice evolved with a better response to therapy, lower tumor growth rate, and intestinal microbiota enriched with *Faecalibacterium*. In addition, they had a higher amount of CD8+ T cells, consistent with the results in human models.

Matson et al. (2018)^73^ evaluated 42 patients with metastatic melanoma, of whom 26 responded to anti PD-1 treatment and 16 had disease progression. The most abundant bacteria among patients in group R were *Bifidobacterium adolescentis, Bifidobacterium longum, Lactobacillus species, Klebsiella pneumoniae, Veillonella parvula, Parabacterioides shite, Collinsella aerofaciens,* and *Enterococcus faecium*, while among NR patients they were *Roseburia intestinalis* and *Ruminococcus obeum*. Then human fecal material was transferred to germ-free mice, followed by inoculation of melanoma cells. They observed that the mice that received fecal material from the R group showed less tumor growth and greater infiltration of effector CD8+ T cells compared with those that received the microbiota from the NR group. The response to anti PD 1 therapy was also consistent with the response of human donors. However, there was a portion that did not have the same intestinal pattern as the donor, and there was no response to therapy. In view of this, they concluded that, although most mice mimic the donor’s response, certain bacteria may have different patterns of expansion and, therefore, generate changes in the recipient’s phenotype.

### Influence of FMT on the response to anti-PD-1/PD-L1 in humans

Based on the recognition of the influence of fecal transplantation associated with immunotherapy on the course of melanoma in mice, clinical trials were carried out with promising results. The trial conducted by Davar et al. (2021)^38^ analyzed the effect of FMT in 15 individuals with melanoma resistant to anti PD 1 therapy. After transplantation, six of the 15 patients (40%) showed an increase in the diversity of the intestinal microbiota (with a predominance of *Ruminococcaceae, Bifidobacteriaceae,* and *Lachnospiraceae*), a greater response to anti PD 1, an increase in CD8+ T cells activation, and a decrease in IL 8 producing myeloid cells. Therefore, it was shown that resistance to immunotherapy was overcome, as these patients, previously unresponsive to anti-PD-1 therapy, showed post-FMT clinical benefit, with tumor reduction and/or long-term disease stability. In addition, FMT has also been shown to modulate the levels of circulating chemical signals, since the R group showed a decrease in multiple cytokines associated with resistance to anti-PD-1 and an increase in biomarkers associated with a beneficial response to treatment. R patients were down regulated in circulating IL-8 as well as in tumor-producing IL-8 myeloid cells; IL-8 has been associated with low anti-PD-1 activity in several cancers, including melanoma.

Similar results were listed in the studies by Youngster et al. (2019)^36^ and Baruch et al. (2021)^39^, and in both there was a post-FMT increase in the infiltration of CD8+ T cells, as well as APCs (CD68+) in the intestine and in the tumor. Both examined therapy-resistant subjects with metastatic melanoma and defined resistance as the inability to achieve a lasting response to anti-PD-1 therapy. In the test performed by Youngster et al. (2019)^36^, three patients (60%) overcame resistance to immunotherapy. Furthermore, in this same study, there was a post-FMT increase in bacteria from the *Paraprevotellaceae* family, which have been associated with responsiveness to treatment, and a significant decrease in β-proteobacteria, which have been linked to resistance to treatment. In the phase 1 clinical trial carried out by Baruch et al. (2021)^39^, patients were treated with vancomycin and neomycin, aiming to eradicate their native microbiota, before receiving the lyophilized solution of fecal material associated with immunotherapy. They observed positive responses in three individuals, two partial and one complete. It is also worth noting that the patients with the highest response rate had an abundance of *Ruminococcus (R. gnavus and R. callidus)* and *Bifidobacterium adolescentis*, considered favorable to immunotherapy, while those with a lower response rate had a microbiota enriched in bacteria from the *Clostridiaceae* family. The researchers also showed an increase in the beneficial immune response.

In the study carried out by Maleki et al. (2020)37, two individuals who had resistant melanoma underwent FMT from two different donors. Both patients showed an improvement in immune response, with an increase in the populations of CD39+ and CD8+ T cells, in addition to a decrease in the levels of dysfunctional PD1+, CD38+, and CD8+ T cells. Patient 1 also displayed stabilization of a large skin lesion.

Some clinical trials aiming to evaluate the efficacy and safety of the ICI-FMT combination are in progress (Table 3).

**Table 3:** Ongoing clinical trials with the application of combined ICI-FMT therapy in cancer patients (www.clinicaltrials.gov) ^74^ .

NCT number	Type of cancer	Number of patients	Intervention	Stage
NCT04521075	Melanoma; CPCNP	50	FMT + Nivolumab	Phase 2
NCT04988841	Melanoma	60	MaaT013 + Ipilimumab + Nivolumab versus placebo + Ipilimumab + Nivolumab	Phase 2
NCT03341143	Melanoma	20	FMT + Pembrolizumab	Phase 2
NCT03772899	Melanoma	20	FMT + Pembrolizumab/Nivolumab	Phase 1
NCT03353402	Melanoma	40	FMT + ICI	Phase 1
NCT04577729	Melanoma	60	Allogeneic FMT + ICI versus autologous FMT + ICI	Not applicable

*FMT: fecal microbiota transplantation; ICI: immune checkpoint inhibitor. Source: Authors (2023).*

Despite the promising results of ICI-FMT therapy, there are still concerns about its long-term safety. In 2019, two independent clinical trials reported that two patients developed bacteremia by extended-spectrum β-lactamase-producing *E. coli* after receiving FMT from the same donor, leading to the death of one of them^75^. This study prompted the FDA to issue a safety bulletin warning of the risk of post-FMT infection. In addition, a recent retrospective cohort study analyzed donor stools and showed that six of the 66 individuals tested (9%) were positive for multidrug-resistant pathogens^76^.

Therefore, periodic screening of donor stools should be performed to strictly limit the spread of organisms that can lead to adverse events, which is especially relevant for immunocompromised patients. Additional clinical investigations, which allow a better understanding of the source, the procedure, and the phenotype of both the recipient and the donor are essential for the success of ICI-FMT combined therapy^27^.

This review presented consistent evidence regarding the use of ICI-FMT therapy in patients with refractory melanoma. However, it is worth mentioning its limitations. Among them, stands out the scarcity of studies that offer relevant data on the application of fecal microbiota transplantation associated with immunotherapy in individuals affected by melanoma. In addition, the few studies found also had limitations, mainly small sample size. Thus, we haighlight the need to conduct new randomized and controlled clinical trials, with a well-defined methodological design, prolonged follow-up time, representative sample, and low risk of bias, to produce robust results and allow a more in-depth analysis of the benefits of this alternative therapy.

## CONCLUSION

FMT has a positive effect on the response of melanoma to ICIs, translated into a significant clinical benefit. However, there is still no consensus regarding the specific bacteria that are associated with a superior response, although certain species belong to phylogenetically related groups. These disagreements can be explained by the use of different genomic sequencing techniques, as well as by geographic influences and dietary variations.

In addition, due to the limited amount of literature on the subject, it is not possible to determine the causal mechanism between the intestinal microbiota and the response to anti-PD-1/PD-L1 drugs. Nonetheless, we postulate that stimulation of responses by T cells against microbial antigens is involved, which in turn aids the response against the tumor. In view of this, new studies aimed at the complete elucidation of the bacteria, the mechanisms involved, and the translation of new evidence to the care practice in oncology are indispensable.
